# *Sargassum muticum* and *Jania rubens* regulate amino acid metabolism to improve growth and alleviate salinity in chickpea

**DOI:** 10.1038/s41598-017-07692-w

**Published:** 2017-09-05

**Authors:** Arafat Abdel Hamed Abdel Latef, Ashish Kumar Srivastava, Hani Saber, Eman A. Alwaleed, Lam-Son Phan Tran

**Affiliations:** 1 Botany Department, Faculty of Science, South Valley University, 83523 Qena, Egypt; 2 Biology Department, College of Applied Medical Science, Turabah Branch 21955, Taif University, Taif, Saudi Arabia; 3 Nuclear Agriculture and Biotechnology Division, Bhabha Atomic Research Centre, Mumbai, 400085 India; 4 Institute of Research and Development, Duy Tan University, 03 Quang Trung, Da Nang, Vietnam; 5 Signaling Pathway Research Unit, RIKEN Center for Sustainable Resource Science, 1-7-22, Suehiro-cho, Tsurumi Yokohama, 230-0045 Japan

## Abstract

The present study evaluates the potential of *Sar* *gassum muticum* (Sar) and *Jan* *ia rubens* (Jan) seaweeds for enhancing growth and mitigating soil-salinity in chickpea (*Cicer arietinum* L.). Under control conditions, Sar and Jan extracts improved chickpea growth which was attributed to their potential for increasing photosynthetic pigments, K^+^ and amino acids, particularly proline, in comparison with water-sprayed control. Upon stress imposition, chickpea growth was reduced in NaCl concentration-dependent manner, and principal component analysis (PCA) revealed Na^+^ accumulation and oxidative damage as major determinants of sensitivity at high salinity. Furthermore, amino acid quantification indicated activation/deactivation of overall metabolism in roots/shoots, as an adaptive strategy, for maintaining plant growth under salt stress. Sar and Jan extract supplementations provided stress amelioration, and PCA confirmed that improved growth parameters at high salinity were associated with enhanced activities of superoxide dismutase and peroxidase. Besides, four key amino acids, including serine, threonine, proline and aspartic acids, were identified from roots which maximally contribute to Sar- and Jan-mediated stress amelioration. Sar showed higher effectiveness than Jan under both control and salt stress conditions. Our findings highlight “bio-stimulant” properties of two seaweeds and provide mechanistic insight into their salt-ameliorating action which is relevant for both basic and applied research.

## Introduction

Seaweeds represent macroscopic and multicellular marine algae that grow naturally at the coastal regions of world and are rich in macro- and micro-nutrients. Since earlier time, seaweed extracts have been used as a fertilizer-additive to reduce the release of chemical fertilizers into environment^1^. Global annual production of seaweeds is around 15 million metric tonnes^2^, with an economic value of 6 billion USD^3^. Chemical composition analysis of seaweeds revealed that they are rich source of hormones and diverse small molecules like proline, betaine, phenolics, flavonoids, sterols and polysaccharides which can modulate multiple cellular processes inside the plants^4^. Furthermore, they also contain chemicals of non-biological origin, such as alginates that can facilitate the aggregation between soil particles, resulting in increased nutrient uptake^5^. For these reasons, paradigm to use seaweed extracts has been shifted from simple fertilizer-additive to a biostimulant for improving plant growth and tolerance to various types of stress^6–9^. Due to the biological origin, seaweed-based formulations are self-degradable, and hence considered eco-friendly.

There are more than 9,000 species of macroalgae which are broadly classified into three main groups based on their pigmentation. For instance, Phaeophyta, Rhodophyta and Chlorophyta represent brown, red and green algae, respectively. *Ascophyllum nodosum* (L.) is the most researched brown alga, and its extract has been commercialized as Acadian^®^ for enhancing different plant growth attributes under normal and stress conditions^10–13^. Recently, *A. nodosum* extract has been used for enhancing oil composition and antibacterial property of mint and basil^14^. Kelpak^®^ is another seaweed concentrate derived from a brown seaweed (*Ecklonia maxima*), and has been demonstrated to act as a biostimulant^15–17^. A novel phlorotannin called Eckol has been isolated from Kelpak^®^ which has been found to have auxin-like activities, and its growth promoting activity has been reported in plants^18–20^. *Sargassum muticum*, commonly known as Japanese wireweed, is a brown seaweed that is invasive to coasts of British Isles, mainland Europe and North America. It has naturally high content of antioxidants, carotenoids and phenols, including the well-known anti-cancer compound fucoxanthin, which makes *S. muticum* important for food, fuel and pharmaceutical industry^21^. Besides, a few red algae, such as *Laurencia obtusa*, *Corallina elongata* and *Jania rubens*, have also been evaluated for their potential to improve plant growth^4, 22^. Seaweeds are naturally adapted to grow under saline environment. Although the exact mechanism behind this salt adaptation is not yet clear, the potential of seaweeds to produce unique combination of metabolites, proteins and phytohormones might have significant contribution. Recently, *Ecklonia maxima* extract has been shown to have salt ameliorating potential in *Cucurbita pepo* under greenhouse conditions^17^. However, not much information is currently available on the salt mitigating potential of seaweed extracts in leguminous crops.

Chickpea (*Cicer arietinum* L.) is the second most important crop, which serves as a rich source of proteins and essential amino acids for humans and animals^23^. Being a legume, it also has the ability to improve soil fertility by fixing atmospheric nitrogen^24, 25^, contributing to sustainable agriculture. However, soil salinity is one of the important environmental constraints which significantly decreases the growth and productivity of this crop worldwide^26, 27^. One of the major reasons behind the negative impacts of salt stress in chickpea is the increased oxidative damage and ionic imbalance^26^. In the present study, we have tested the potential of brown (*Sargassum muticum*) and red (*Jania rubens*) seaweed extracts for improving growth and ameliorating salt stress in chickpea at vegetative stage. On the basis of clustering and PCA analysis of different growth and biochemical attributes, the ability of these seaweeds to maintain Na^+^ and K^+^ homeostasis and reduce oxidative damage in chickpea was found as major contributors behind the ameliorative effects of the extracts derived from both seaweeds. However, *S. muticum* was found to be a better choice over *J. rubens*. Our findings further extend the applications of these seaweeds in the field of agriculture.

## Methods

### Seaweed Collection and Extract Preparation

Brown (*Sargassum muticum*) and red (*Jania rubens*) algae were collected in April-May 2014 from shallow water along the shore of Red Sea, Safaga, Egypt. The identification of species was carried out using standard taxonomic practices (Edwards *et al*. 2012). The collected samples were washed with sterilized water to remove debris, dried initially for 7 days under sunlight and then heated at 80 °C for 24 h. Dried seaweeds were ground to fine powder using electric mill, and then the powder was boiled with water at 1:1 ratio (w/v) for 2 h. The homogenate was filtered using Whatman No. 2 filter paper, and the extract was stored in amber colored bottles at 4 °C. ***Sar*** *gassum muticum* (Sar) and ***Jan*** *ia rubens* (Jan) extracts were further diluted to make different concentrations, including 0, 0.1, 0.5, 1, 1.5, 2 and 2.5%, and then used in preliminary evaluation of growth response of chickpea seedlings. On the basis of the preliminary optimal growth test, 1% concentration of the extracts containing 0.1% Tween-20 (at final concentration) was selected for further studies.

### Plant Materials, Growth Conditions and Treatments

The entire experiment was conducted on chickpea (*Cicer arietinum* L.) using pot culture in the wire-house experimental farm of South Valley University, Qena city, Egypt. Plants were grown under natural conditions of temperature, light and humidity during the winter season of 2015. Chickpea seeds (equi-sized and -weight) were surface-sterilized with 0.1% mercuric chloride for 5 minutes and then rinsed 3 times with sterile distilled water. Seeds were germinated on plastic pots (10 seeds/pot) filled with 2 kg of dried soil. After 7 days, only five healthy plants with similar size were kept in each pot. A total three set of pots were prepared (9 pots/set). Set-1 was irrigated with water and served as control. Set-2 and set-3 were irrigated with 50 and 150 mM NaCl, respectively, from 7 days after sowing. Three pots from each set were foliar-sprayed with water (denoted as WS for water-sprayed), Sar or Jan extracts at days 4^th^ and 11^th^ (11- and 18-day-old plants, respectively) after irrigation with NaCl solution. At day 35^th^, plants were harvested for determination of various parameters.

### Plant Growth Parameters and Pigment Contents

For growth assessment, root and shoot lengths were measured manually with a ruler. Subsequently, the samples were dried at 80 °C for 2–4 days until they achieved constant dry weight (DW) for determination. As for the pigments, chlorophyll a and b (Chl a and Chl b) and carotenoid contents were estimated in leaves using previously described methods^28^.

### Sugar and Total Phenol Contents

The contents of total soluble sugars^29^ and total phenols^30^ were quantified in dried root and shoot samples using standard methods reported previously.

### Measurement of Na^+^ and K^+^ Contents

The contents of Na^+^, K^+^ and Ca^2+^ in dried roots and shoots were determined using flame photometry as per the method described previously^31^.

### *Assays for* Oxidative Damage, ROS Content and Activities of Enzymatic Antioxidants

The oxidative damage in fresh leaves was estimated in the form of malondialdehyde (MDA) content using thiobarbituric acid (TBA) method^32^. The H_2_O_2_ contents in fresh leaf samples were measured according to previously published method^33^. For the measurement of the activities of enzymatic antioxidants, leaves were grounded in liquid N_2_, and total proteins were extracted as described^34^. The activities of superoxide dismutase (SOD; EC 1.15.1.1), catalase (CAT; EC 1.11.1.6), peroxidase (POD; EC 1.11.1.7) and ascorbate peroxidase (APX; EC 1.11.1.11) were determined according to the respective methods published previously by refs 35–38.

### Profiling of Amino Acids

The amino acid contents in dried roots and shoots (3 biological replicates) were determined in amino acid laboratory at the National Center of Radiation Research and Technology (NCRRT), Cairo, Egypt, using previously described method^39^.

### Statistical Analysis

The mean values from control and treated samples were separated using Duncan’s multiple range test (DMRT) at the significant level of *p* < 0.05 using SPSS (version 21.0; IBM Corp., Armonk, NY, USA). Data presented are means ± standard errors (SEs) of three replicates. The normalized mean values were used for clustering analysis and heatmap was generated using MeV software (http://mev.tm4.org/). Principal component analysis (PCA) was performed using Origin 17 (OriginLab, Northampton, MA, USA), and the first two components (PC1 and PC2) explaining the maximum variance in the datasets were used to make biplots.

## Results

### Foliar applications of seaweed extracts improved plant growth and photosynthetic pigments under normal and saline soil conditions

**Figure 1:**
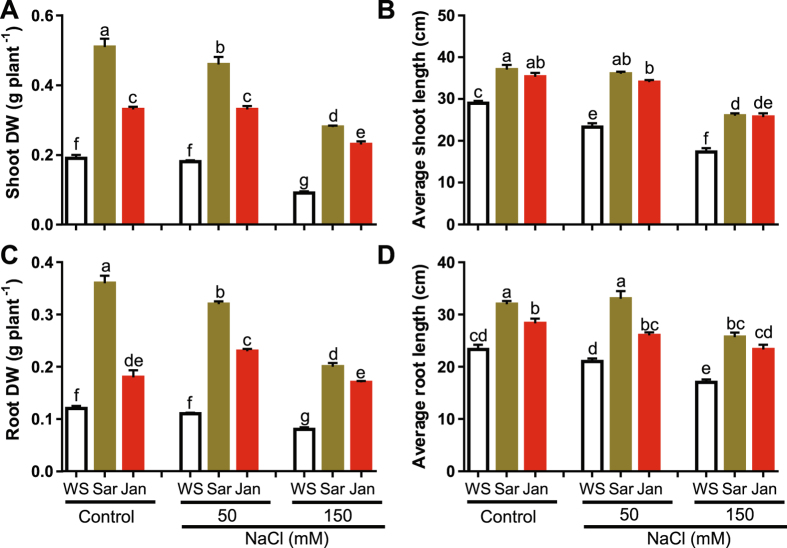
Effects of Sar and Jan extract applications on plant growth attributes of 35 day-old chickpea plants under normal and soil-saline conditions. (**A**) shoot dry weight (DW), (**B**) shoot length, (**C**) root DW and (**D**) root length. Data represent means and standard errors (error bars) of three biological replicates. Different letters indicate significant difference (*p* < 0.05), according to a Duncan’s multiple range test. WS, Sar and Jan represent plants receiving foliar application of water, Sar and Jan extracts, respectively.

Sar and Jan applications significantly improved the vegetative growth of plants under both control and saline-soil conditions. Under control conditions, shoot DW and length were increased by 165 and 28% in Sar- and 70 and 22% in Jan-sprayed plants (Fig. 1A,B), while root DW and length were increased by 212 and 37% in Sar- and 58 and 70% in Jan-sprayed plants, respectively, as compared with that of non-stressed water-sprayed WS plants (Fig. 1C,D).

At 50 mM NaCl, no significant reduction in the biomass of both shoots and roots was observed, but shoot and root lengths of stressed WS plant were decreased by 20 and 10%, respectively as compared with that of unstressed WS control (Fig. 1B and D). At 150 mM NaCl, shoot DW and length of salt-treated WS plant were reduced by 52 and 40% and root DW and length were decreased by 34 and 27%, respectively, as compared with that of untreated WS control (Fig. 1A–D). Foliar applications of Sar and Jan significantly ameliorated the growth phenotype under NaCl treatment, and the better effects were observed in terms of biomass accumulation. Sar increased shoot and root DWs by 163 and 180% (at 50 mM) and 207 and 158% (at 150 mM), respectively, while Jan extract increased the respective parameters by 87 and 105% (at 50 mM) and 151 and 128% (at 150 mM), as compared with that of stressed WS plants (Fig. 1A and C). The increased biomass accumulation was also reflected in terms of increased root and shoot lengths of Sar- and Jan-sprayed plants as compared with that of WS plants (Fig. 1B and D).

**Figure 2:**
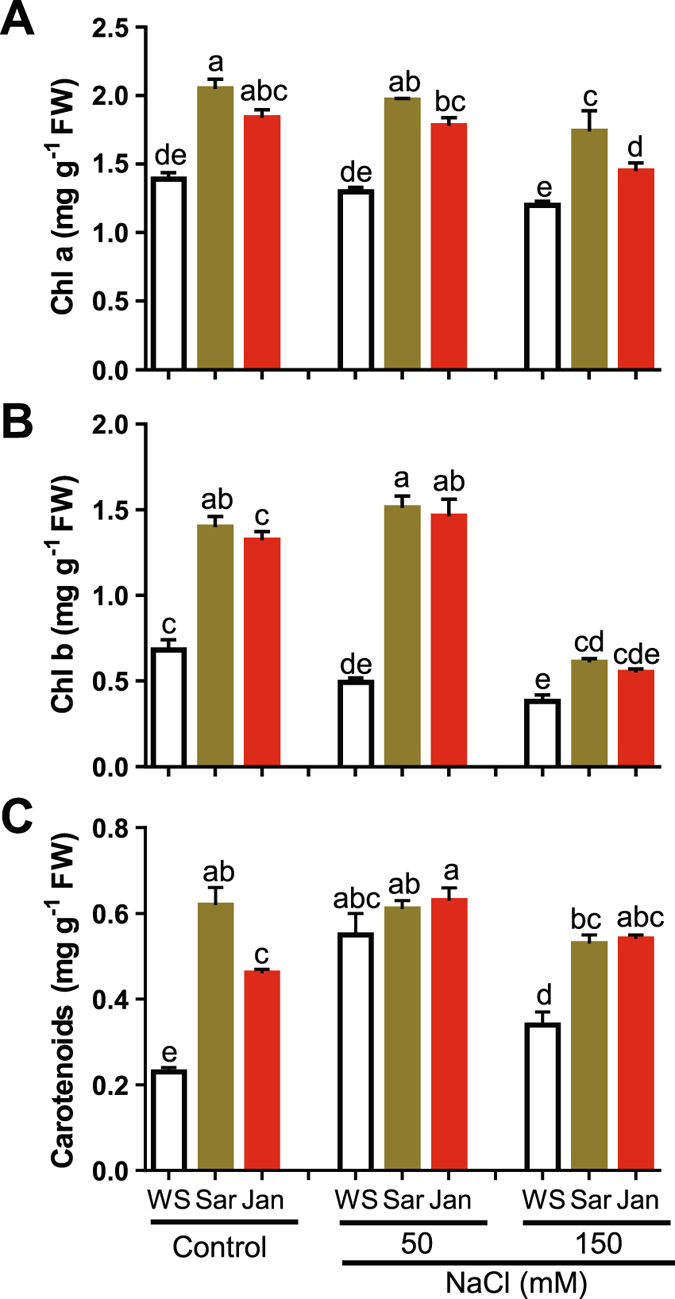
Effects of Sar and Jan extract applications on biosynthesis of photosynthetic pigments in 35 day-old chickpea plants under normal and soil-saline conditions. (**A**) chlorophyll a (Chl a), (**B**) chlorophyll b (Chl b), and (**C**) carotenoid contents. Data represent means and standard errors (error bars) of three biological replicates. Different letters indicate significant difference (*p* < 0.05), according to Duncan’s multiple range test. WS, Sar and Jan represent plants receiving foliar application of water, Sar and Jan extracts, respectively. FW, fresh weight.

The data of growth-attributing parameters were positively correlated with those of photosynthetic pigments under different treatments. In control plants, Sar application increased the levels of Chl a, Chl b and carotenoids by 48, 105 and 168%, while Jan increased their levels by 32, 95 and 101%, respectively, as compared with that of control WS plants (Fig. 2A–C). Imposition of NaCl stress on WS plants significantly decreased Chl b by 28 and 45%, whereas increased carotenoids by 138 and 46% at 50 and 150 mM NaCl, respectively, as compared with that in non-stressed WS plants (Fig. 2B,C). No significant change in Chl a was observed in stressed WS plants at any of the tested salt concentrations (Fig. 2A). The supplementation of seaweed extracts significantly increased the levels of these pigments under salt stress. At 50 mM NaCl, Chl a was increased by 51 and 37%, while Chl b was increased by 210 and 199% in salt-treated plants sprayed with Sar and Jan, respectively, as compared with that of salt-treated WS plants (Fig. 2A,B). No significant difference in carotenoid contents was observed in Sar- or Jan-sprayed plants at 50 mM NaCl concentration, as compared to that of respective WS plants (Fig. 2C). At 150 mM, Chl a, Chl b and carotenoids were increased by 45, 62 and 57% in salt-stressed plants sprayed with Sar and 21, 45 and 60 in salt-treated plants sprayed with, respectively, in comparison with respective salt-treated WS control (Fig. 2A–C).

### Differential accumulations of soluble sugars and total phenols upon supplementation of seaweed extract

**Figure 3:**
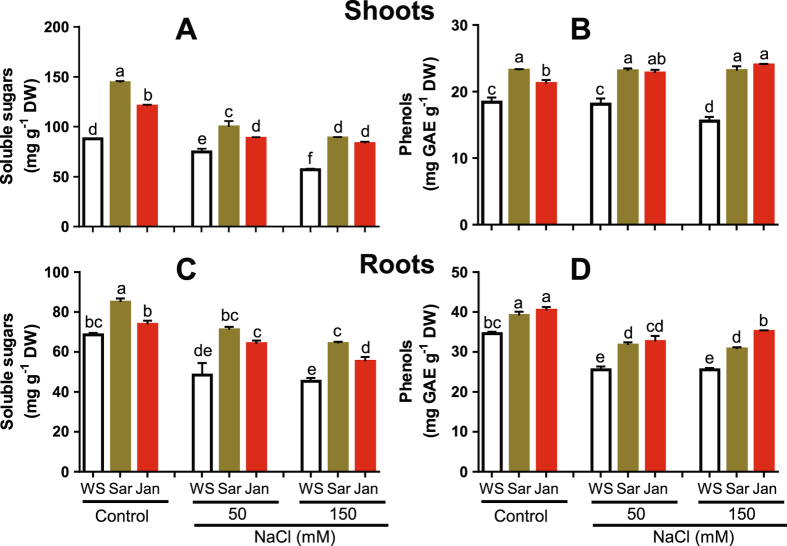
Effects of Sar and Jan extract applications on various metabolite contents in 35 day-old chickpea plants under normal and soil-saline conditions. (**A**) soluble sugar contents in shoots, (**B**) phenol contents in shoots, (**C**) soluble sugar contents in roots (**D**) phenol contents in roots. Data represent means and standard errors (error bars) of three biological replicates. Different letters indicate significant difference (*p* < 0.05), according to a Duncan’s multiple range test. WS, Sar and Jan represent plants receiving foliar application of water, Sar and Jan extracts, respectively. DW, dry weight; GAE, gallic acid equivalent.

Under control conditions, applications of Sar and Jan extracts significantly increased the levels of soluble sugars and total phenols in both roots and shoots, with the higher increase being observed in shoots. In Sar-treated shoots, soluble sugars and total phenols were increased by 64 and 26%, while in Jan-treated shoots these parameters were increased by 37 and 15%, respectively, relative to that in WS plants (Fig. 3A,B).

Under NaCl stress conditions, the levels of soluble sugars and total phenols were decreased in salt-treated WS plants, as compared with that in non-stressed WS control (Fig. 3). Spraying salt-treated plants with Sar and Jan avoided these reductions. At 50 mM NaCl, levels of soluble sugars and phenols were increased by 47 and 24% and 32 and 28% in roots of Sar-sprayed and Jan-sprayed plants, respectively, while, at 150 mM NaCl, these values were increased by 41 and 20% and 22 and 38% in roots subjected to Sar and Jan treatment, respectively, as compared with that in roots of stressed plants exposed to WS treatment (Fig. 3C,D). As for shoots under 50 mM NaCl stress, soluble sugar and total phenol contents were increased by 33 and 28% in Sar and 18 and 26% in Jan treatment, respectively, as compared with that of WS treatment. At 150 mM NaCl, soluble sugar and phenol levels were increased by 56 and 49% in Sar and 46 and 54% in Jan-treated shoots, respectively, relative to that of WS shoots (Fig. 3A,B).

### Application of seaweed extracts modulates accumulation of Na^+^ and K^+^ ions

**Figure 4:**
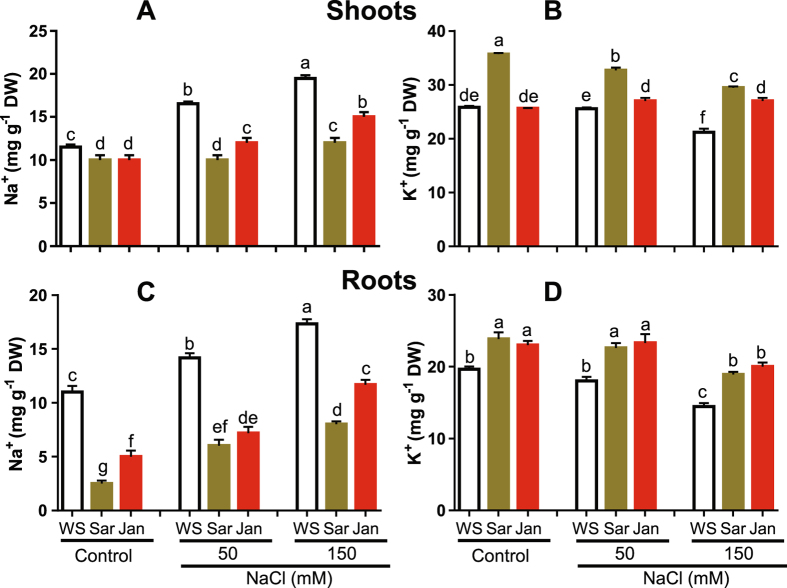
Effects of Sar and Jan extract applications on ion accumulations in 35 day-old chickpea plants under normal and soil-saline conditions. (**A**) shoot-Na^+^, (**B**) shoot-K^+^, (**C**) root-Na^+^ and (**D**) root-K^+^ contents. Data represent means and standard errors (error bars) of three biological replicates. Different letters indicate significant difference (*p* < 0.05), according to a Duncan’s multiple range test. WS, Sar and Jan represent plants receiving foliar application of water, Sar and Jan extracts, respectively. DW, dry weight.

Under non-stressed conditions, Na^+^ levels in shoots and roots were decreased by 13 and 77% by Sar and 13 and 55% by Jan treatment, respectively, in comparison with WS treatment (Fig. 4A and C). Simultaneously, K^+^ levels in shoots and roots of Sar-treated plants were increased by 38 and 21%. However, increased K^+^ was limited to roots of in Jar-sprayed plants only, with 17% over that in roots of WS plants (Fig. 4B and D).

In general, NaCl-treated WS plants showed increased Na^+^ and decreased K^+^ levels. Na^+^ levels in shoots and roots were augmented by 43 and 29% at 50 mM NaCl; while, 69 and 58% at 150 mM NaCl, respectively, as compared with that in non-treated control WS plants (Fig. 4A and C). Significant decrease in K^+^ level was observed only at 150 mM NaCl, by 18 and 26% in shoots and roots of salt-stressed WS plants, respectively, relative to that in unstressed WS control (Fig. 4B and D). Sar and Jan supplementations reduced the extent of Na^+^ accumulation and maintained higher K^+^ levels by almost similar extent in both the tested NaCl concentrations. At 150 mM NaCl, Na^+^ levels in shoots and roots were reduced by 38 and 54% by Sar and 23 and 33% by Jan treatment, respectively, as compared with that obtained by WS treatment (Fig. 4A and C). Under the same 150 mM NaCl conditions, K^+^ levels in shoots and roots were increased by 39 and 31% by spraying with Sar and 28 and 38% with Jan, respectively, in comparison with WS treatment (Fig. 4B and D).

### Oxidative Damage, ROS Accumulation and Antioxidant Enzyme Activities under Different Treatments

**Figure 5:**
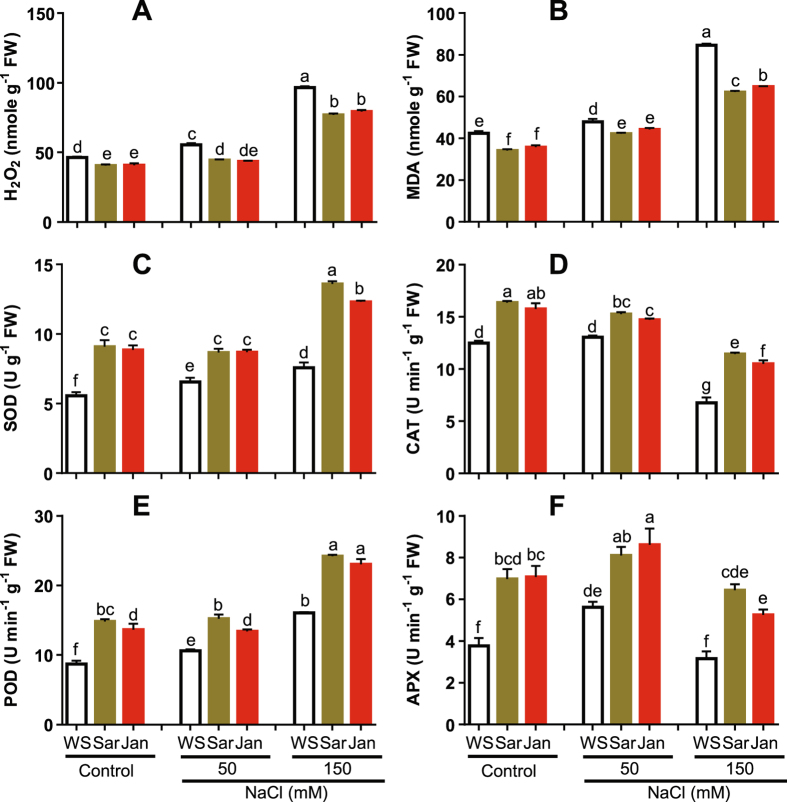
Effects of Sar and Jan extract applications on contents of malondialdehyde (MDA) and hydrogen peroxide (H_2_O_2_), and activities of antioxidant enzymes in 35 day-old chickpea plants under normal and soil-saline conditions. (**A**) H_2_O_2_ content, (**B**) MDA content, (**C**) superoxide dismutase (SOD), (**D**) catalase (CAT), (**E**) peroxidase (POD) and (**F**) ascorbate peroxidase (APX) activities. Data represent means and standard errors (error bars) of three biological replicates. Different letters indicate significant difference (*p* < 0.05) according to a Duncan’s multiple range test. WS, Sar and Jan represent plants receiving foliar application of water, Sar and Jan extracts, respectively. FW, fresh weight.

Under normal growing conditions, H_2_O_2_ and MDA levels were reduced by 12 and 20% in plants treated with Sar and 12 and 16% in plants sprayed with Jan, respectively, relative to that in WS plants (Fig. 5A,B). SOD, CAT, POD and APX activities were increased by 63, 31, 70 and 86% in Sar- and 59, 26, 56 and 88% in Jan-treated plants, respectively as compared with that in WS plants (Fig. 5C–F).

Upon exposure of plants to NaCl stress, H_2_O_2_ and MDA levels were increased in a dose-dependent manner with the maximum increase of 108 and 99%, respectively, being observed in WS plants exposed to 150 mM NaCl compared with that obtained from non-stressed control WS plants (Fig. 5A,B). At 50 mM NaCl, CAT activity was relatively unchanged, while SOD, POD and APX activities were increased by 17, 22 and 49%, respectively, over unstressed control. At 150 mM NaCl, SOD and POD activities were increased by 36 and 84%, while CAT and APX activities were decreased by 46 and 16%, respectively, in WS plants as compared with that of non-stressed control. Sar and Jan extracts significantly increased enzymatic antioxidant activities, and consequently reduced H_2_O_2_ and MDA levels at both the tested NaCl concentrations. However, the difference was more prominent at 150 mM NaCl. The H_2_O_2_ and MDA levels at 150 mM NaCl were decreased by 20 and 27% in Sar-treated and 18 and 23% in Jan-treated plants in comparison with WS plants (Fig. 5A,B). As for the antioxidant enzymes, SOD, CAT, POD and APX activities were increased by 79, 69, 51 and 104% in Sar-sprayed and 62, 55, 43 and 66% in Jan-sprayed plants pre-treated with 150 mM NaCl, respectively, as compared with that of WS salt-treated plants (Fig. 5C,D).

### Understanding Treatment-variable Interactions through Hierarchical Clustering and PCA Analysis

**Figure 6:**
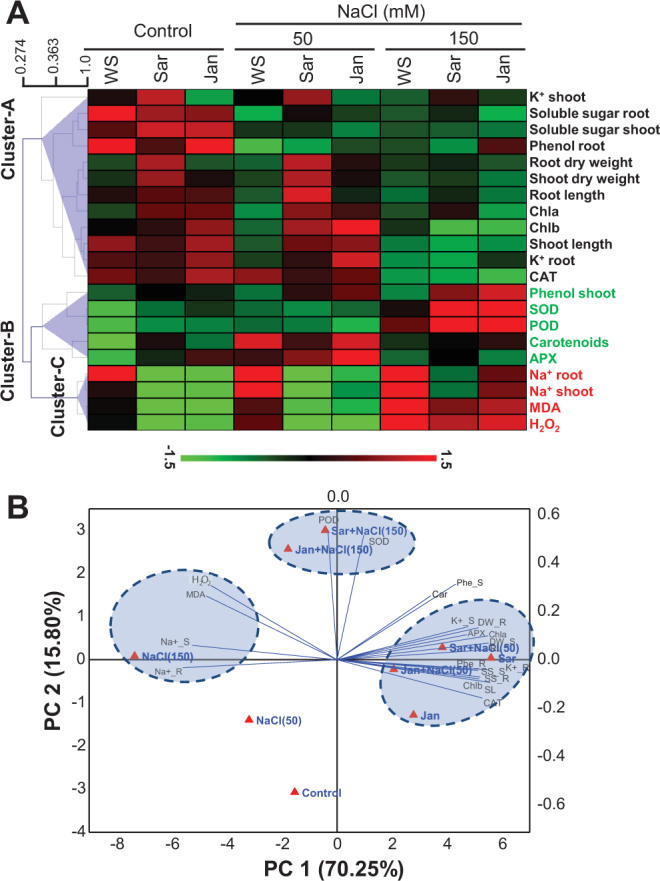
Hierchial clustering and principal component analysis (PCA) to understand treatment-variable relationships in chickpea plants. (**A**) The mean values of different parameters were normalized and clustered. Variables associated with three major clusters A, B and C are demarcated with black, green and red color, respectively. (**B**) The entire data were analyzed using PCA. The lines originating from central point of biplots indicate positive or negative correlations of different variables; where their closeness indicates correlation strength with particular treatment. The variables included DW_R (root dry weight), DW_S (shoot dry weight), RL (root length), SL (shoot length), Chl a (chlorophyll a), Chl b (chlorophyll b), Car (carotenoids), SS_R (root-soluble sugars), SS_S (shoot-soluble sugars), Phe_R (root-phenols), Phe_S (shoot-phenols), Na^+^_R (root-Na^+^), Na^+^_S (shoot-Na^+^), K^+^_R (root-K^+^), K^+^_S (shoot-K^+^), SOD (superoxide dismutase), CAT (catalase), POD (peroxidase), APX (ascorbate peroxidase), MDA (malondialdehyde) and H_2_O_2_ (hydrogen peroxide). Refer to Supplementary Table 1 for cumulative PCA scores for each treatment. WS, Sar and Jan represent plants receiving foliar application of water, Sar and Jan extracts, respectively.

The clustering analysis of the entire data, representing average mean values of various parameters in WS, Sar- and Jan-sprayed plants under control and two conditions of NaCl stress, yielded three distinct clusters (Fig. 6A). Cluster-A, which mainly represents growth attributes, contents of photosynthetic pigments, K^+^ level, contents of soluble sugars and phenols, and activity of enzymatic antioxidants like CAT, showed decreasing trend in WS plants treated with either 50 or 150 mM NaCl as compared with non-stressed control. Sar- and Jan-treated plants showed complete/partial restoration of this cluster only at 50 mM NaCl as compared with that of stressed WS plant. Cluster-B represents activities of enzymatic antioxidants, such as SOD, POD and APX, carotenoid content and phenol levels of shoots. These parameters were mainly altered at 150 mM NaCl in WS plants compared with non-stressed WS plants, and partial/complete restoration was also maintained in Sar- and Jan-treated plants. Cluster-C represents Na^+^, MDA and H_2_O_2_ levels, which showed concentration-dependent increase in WS plants in response to NaCl stress as compared with that of control, while Sar- and Jan-treated plants showed resilience at both 50 and 150 mM NaCl when compared with stressed WS plants. In order to understand the association of these clusters with different treatments, PCA analysis was then performed. Results revealed that WS plants at 150 mM NaCl were associated with Cluster-C variables (Fig. 6B). Under control and 50 mM NaCl, Sar- and Jan-treated plants were associated with variables of Cluster-A variables, while at 150 mM NaCl they were associated with those of Cluster-B such as SOD and POD activities. WS plants did not show significant association with any parameters under control and at 50 mM NaCl (Fig. 6B). For PCA score under different treatments refer Supplementary Table 1.

### Amino acid Based Compositional Analysis under Different Treatments

**Figure 7:**
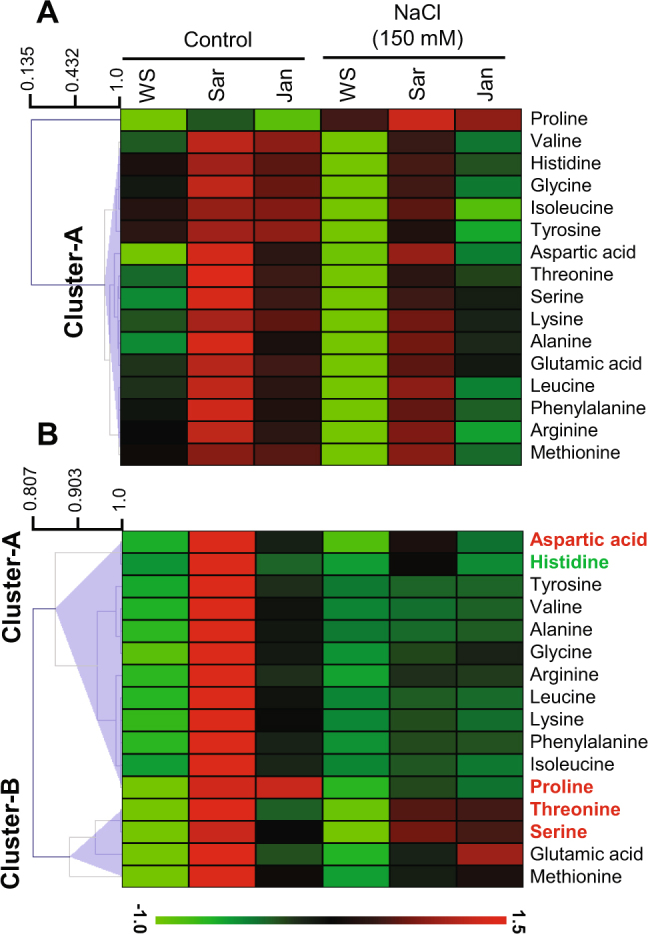
Profiling of different amino acids in 35 day-old chickpea plants under normal and soil-salinity conditions. (**A**) shoot and (**B**) root amino acid patterns. Heat-map represents the normalized mean values of three independent biological replicates. In roots, key amino acid showing more than 1.5 fold increase in both Sar and Jan treated plant under 150 mM NaCl as compared with stressed WS plants are marked as bold red while that increased under Sar alone is marked as bold green. Refer to Supplementary Table 2 for absolute changes in amino acid level under different treatments. WS, Sar and Jan represent plants receiving foliar application of water, Sar and Jan extracts, respectively. FW, fresh weight.

Under control conditions, levels of different amino acids were increased upon application of seaweed extracts, and the extent of increase was found to be higher with Sar than Jan extract (Fig. 7). The major increase was observed in roots in which average amino acid levels in Sar and Jan treatment were increased by 5.2- and 2.7-fold, respectively, as compared with that of WS control roots. Of different amino acids, proline level was maximally increased by 14.2- and 13.8-fold in Sar- and Jan-treated roots, respectively, as compared with that of WS control plants (Supplementary Table 2).

Upon 150 mM NaCl imposition, a contrasting amino acid pattern was observed in different organs. The average amino acid level in roots and shoots of WS plant was increased and decreased by 1.4- and 0.6-fold, respectively, as compared with that of WS plant obtained under control conditions, with an exception of proline which was increased by 1.4 fold in shoots. Supplementations of Sar and Jan extracts at 150 mM NaCl conditions increased the amino acid levels in both roots and shoots; however, the effects were more pronounced in shoots than roots. Like control conditions, Sar was found to be more effective even at 150 mM NaCl conditions which was evident by a higher increase in average amino acid levels in Sar (2.4-fold) than Jan (1.7-fold) treatment, as compared with that of stressed WS plants. Additionally, using 1.5-fold as a cut-off criterion, four “key” amino acids, including proline, serine, threonine and aspartic acid, have been identified to be increased in both Sar- and Jan-treated roots as compared with that of respective WS under NaCl stress conditions. Besides, histidine was also noted to have an 1.8-fold increase only in Sar-treated roots (Supplementary Table 2).

## Discussion

Seaweed extracts are considered to have unique source of nutrients, hormones and small metabolites^4, 5, 18, 40^. *S. muticum* and *J. rubens* are the most common types of brown and red marine algae, respectively, present at the Red sea coast of Egypt. Considering their natural adaptation to survive and grow under saline environment, a pilot-scale pot experiment was conducted to evaluate and compare the potential of Sar and Jan extracts to improve chickpea growth and ameliorate salt stress effects on chickpea performance at vegetative stage. Additionally, the responses of chickpea plants to two different doses of soil salinity was also examined at physiological and biochemical levels.

### Sar has higher potential than Jan in improving plant growth under non-stressed conditions

The applications of Sar and Jan extracts significantly improved the growth of chickpea plants, in terms of biomass accumulation and height, under control conditions (Fig. 1). PCA analysis clearly depicted the close association of these growth attributes with photosynthetic pigment contents, soluble sugars and K^+^ accumulation (Fig. 6B), suggesting an overall metabolism is accelerated in response to Sar and Jan applications. However, PCA score in Sar- (PC1:5.59; PC2:0.04) was higher than Jan-treated (PC1:2.78; PC2:−1.28) plants (Supplementary Table 1). Furthermore, most of the growth and biochemical parameters were increased to a lesser extent in Jan- than Sar-treated plants (Fig. 6A), particularly K^+^ level in shoots, which was remarkably increased specifically by Sar treatment (Fig. 4B). All these findings corroborate better potential of Sar over Jan extract in improving chickpea growth under control conditions. This was further substantiated by greater increase in average amino acid contents in Sar- than Jan-treated plants, especially in roots (Fig. 7; Supplementary Table 2). Although the extracts were applied through foliar spraying, the major effects on amino acid levels were observed in roots than shoots under control conditions, suggesting the involvement of systemic signaling behind Sar- and Jan-mediated growth enhancement. Earlier, hormones, such as salicylic acid, cytokinins and auxins that can mediate shoot-to-root or *vice versa* communication, have been identified from various seaweed extracts^40^. Of different amino acids in roots, maximum increase was noted in proline level upon Sar and Jan applications, which clearly indicated that proline, apart from its well established function as stress-protectant^41^, is also a key contributor for improving growth. The growth enhancement effect of proline is mainly due to its role in regulating G2/M-specific *CYCLINB1;1* gene expression and providing hydroxyproline-rich glycoproteins that serve as structural constituents of cell wall^42, 43^. Surprisingly, even under control conditions, enzymatic antioxidant activities were increased, resulting in decreased H_2_O_2_ and MDA contents in Sar- and Jan-treated plants, as compared with that in WS plants (Fig. 5). Given the multifaceted role of ROS in plant growth and development^44^, lowering ROS below basal level might reduce growth. However, the study was performed in natural climatic conditions of wire-house, where devoid of stress could not be completely expected; hence, reduction in ROS below control level did not cause any growth retardation in Sar- and Jan-treated plants. Thus, *S. muticum* and *J. rubens* represent promising candidates that could be exploited further to identify novel systemic regulators with potential to boost metabolism and enhance plant growth through modulating proline level. Additionally, these seaweed extracts could serve as an alternative resource to genetic methods for enhancing endogenous proline levels and overall plant growth.

### Physiological and biochemical responses of chickpea plants at different salt concentrations

In order to understand the association between different physiological and biochemical parameters and salt sensitivity, the responses of chickpea plants were assessed at 50 and 150 mM NaCl concentrations at vegetative stage. The negative impacts of NaCl stress on chickpea plants are known and involve different processes, such as reduced water availability, stomata closure, disturbed gaseous exchange, reduced photosynthetic efficiency and energetics, decreased photoassimilates and enhanced ROS production^26, 45, 46^. In the present study, we performed PCA analysis on different variables, and results revealed that Cluster-C, which included Na^+^ accumulation and H_2_O_2_ and MDA contents, was closely associated with salt-sensitive phenotype observed at 150 mM NaCl (Fig. 6). Recently, using a set of contrasting genotypes, it was proposed that NaCl sensitivity in chickpea was mainly determined by Na^+^ level, and, more or less, independent of osmotic components and Cl^−^ toxicity^47^. Although this information provided strength to our conclusion; however, we conceptually advance this point and propose that along with reduced Na^+^ accumulation, well co-ordinated antioxidant defence is equally important to avoid NaCl stress sensitivity in chickpea plants. For instance, cluster-B variables like SOD and POD activities are increased at 150 mM NaCl; however, they are not co-ordianted with CAT and APX activities; and hence, increased oxidative damage was observed (Fig. 5A,B), resulting in reduced plant growth (Fig. 1). Since PCA analysis revealed better association of Na^+^ accumulation and oxidative damage parameters with the higher NaCl concentration (150 mM); therefore, amino acid profiling in WS chickpea plants was performed at 150 mM NaCl. Interestingly, an opposite amino acid pattern was observed in roots and shoots which clearly indicated distinct metabolic states in these organs in response to salt stress conditions. Because the treatment duration for the present study was for 28 days, overall increase and decrease in average amino acid contents in roots and shoots, respectively, might be considered as an adaptive strategy to activate and deactivate amino acids-related metabolic network(s) in roots and shoots, respectively, so that plants can survive the long-term salt exposure. An active metabolism in roots is needed to avoid an excessive salt uptake and to maintain plant nutritional status, while deactivation of metabolism in shoots reduces the transpirational pull which provides further help to plants for controlling Na^+^ uptake. Such an activation/deactivation of root/shoot amino acid metabolism has also been demonstrated under other abiotic stresses, such as heat and drought^48, 49^. In shoots, proline was identified as the only amino acid whose level was increased and not decreased in WS plants under 150 mM NaCl, which might be related to several important functions of proline, such as osmolyte and antioxidants^50^. Thus, the results not only highlight the major changes responsible for salt-sensitive phenotype of chickpea but also provide insight into their adaptive mechanisms under long-term salinity stress.

### Sar and Jan extracts ameliorate salt-sensitive phenotype through modulation of ionic balance and oxidative damage

The foliar application of either Sar or Jan extract significantly ameliorated the negative impacts of NaCl stress. However, like control conditions, effectiveness of Sar was found to be better than Jan, as indicated by its higher PCA score at both 50 and 150 mM NaCl concentrations (Supplementary Table 1). The ameliorative actions of Sar and Jan were attributed to enhanced levels of photosynthetic pigments like Chl a, Chl b and carotenoids (Fig. 2), indicating significant recovery towards NaCl stress-induced damages on photosynthetic efficiency. Improved photosynthesis was supported through assimilation as indicated by increased metabolites, including soluble sugars. Besides, phenolic content was also found higher in Sar- and Jan-treated plants under NaCl stress, as compared with that in WS plants. Increased metabolites are essential to provide energy needed to activate various salinity defence strategies. Additionally, these metabolites could also serve as compatible solutes^51^, thereby facilitating NaCl stress amelioration. One of the key defence mechanisms against NaCl stress is Na^+^ extrusion^52^, which was also observed by Sar and Jan extract applications (Fig. 4A and C), suggesting active roles of Sar and Jan extracts in Na^+^ extrusion. The Na^+^ reduction was concomitant with increased K^+^ in Sar- and Jan-treated plants in comparison with water-treated control, which helps avoid the symptoms associated with salt stress-induced K^+^ deficiency (Fig. 4B and D). The imposition of soil salinity also increased ROS production due to misleading of electrons to oxygen in electron transport chain system of mitochondria and chloroplast^53^. The generated ROS are efficiently detoxified through enzymatic and non-enzymatic antioxidants; however, lack of coordination in detoxification machinery leads to increased ROS levels and oxidative damage in plants^54^. Applications of Sar and Jan extracts efficiently reduced the levels of H_2_O_2_, one of the important ROS produced under NaCl stress, and MDA (an indicator of lipid peroxidation), indicating overall reduction of oxidative damage (Fig. 5). This positive effect was attributed to a co-ordinated increase in the activities of the four tested enzymatic antioxidants SOD, CAT, APX and POD, and levels of metabolites like soluble sugars^55^ and phenolics^56^, which are known to act as ROS scavengers. In addition, proline, which is a potent ROS scavenger^57^, was also found to be increased in both Sar- and Jan-treated plants relative to that of WS plants at 150 mM NaCl (Fig. 7). Thus, Sar- and Jan-mediated amelioration of NaCl-induced toxicity was associated with multiple mechanisms, including enhanced photosynthesis, increased metabolite accumulation, balanced ionic content and improved antioxidant defence.

Clustering and PCA analyses were performed to identify key components responsible for Sar- and Jan-mediated soil salinity amelioration. With the increase in NaCl concentration from 50 to 150 mM, the association of Sar- and Jan-treated plants was shifted from Cluster-A variables to Cluster-B variables. Moreover, at 150 mM NaCl, SOD and POD were identified as key variables for shaping plant phenotype (Fig. 6B). SOD, considered as the first line of defence against ROS, is responsible for converting superoxide radicals (O_2_^•−^) into H_2_O_2_^58^, and POD represents Class III peroxidases which functions to detoxify H_2_O_2_^59^. Unlike WS plants whose diminutive phenotype under 150 mM NaCl was associated with increased H_2_O_2_ and MDA levels, increased activities of SOD and POD in Sar- and Jan-treated plants reduced the extent of oxidative damage, ultimately resulting in significant growth amelioration.

Considering seaweeds as a rich source of proteins^60^, the levels of different amino acids were profiled in plants supplemented with Sar and Jan extracts. In roots, after applying a criterion of 1.5-fold as a cut-off, we identified serine, threonine, proline and aspartic acid as key amino acids responsible for Sar- and Jan-mediated ameliorations under 150 mM NaCl stress conditions (Fig. 7). Serine and threonine containing proteins, including plant-specific SnRKs (sucrose nonfermenting-related kinases), play fundamental roles in mediating stress signalling through phosphorylation/dephosphorylation mechanisms^61^. Proline has multifaceted actions such as osmolyte, antioxidant and energy source^50^, while aspartate serves as an intermediate to support the synthesis of other amino acids, including lysine, threonine, isoleucine and methionine^62^. In addition, histidine, which was specifically increased in Sar-treated plants, also represent an important constituent of proteins responsible for stress sensing^63^. Thus, the association of key amino acids with stress signaling clearly indicate that Sar and Jan extracts do not just function as aminoacid supplements but act as “bio-stimulants”, boosting plant signaling under stress conditions. Furthermore, most of the amino acids remained comparatively elevated in Sar- and Jan-treated shoots at 150 mM NaCl than those in stressed WS shoots, verifying the lack of metabolic-deactivation effects in shoots (Fig. 7). Since the extent of increase of different amino acids was comparable in Sar- or Jan-treated shoots under salt stress, we could not identify top-ranked amino acids responsible for Sar- or Jan-mediated stress ameliorative effects in shoots. In future, deeper compositional analyses of Sar and Jan extracts are required to identify exact causative agent(s) responsible for different biochemical changes and stress amelioration.

## Conclusion

The present study was conducted to evaluate the potential of a brown ***Sar*** *gassum muticum* (Sar) and red ***Jan*** *ia rubens* (Jan) seaweed extracts for enhancing growth and ameliorate negative effects of soil salinity on chickpea at vegetative stage. Under control conditions, Sar and Jan enhanced chickpea growth, which was attributed to the increased levels of photosynthetic pigments, soluble sugars and amino acids, majorly proline. The salinity-sensitive phenotype of chickpea, particularly at 150 mM NaCl, was found to be associated with increased accumulation of Na^+^ and oxidative damage. Besides, amino acid profiling revealed metabolic activation and deactivation in roots and shoots, respectively, which help plants grow during exposure to long-term NaCl stress. Applications of Sar and Jan extracts significantly ameliorated the negative impacts of soil salinity through multiple mechanisms, including balanced ionic content and improved antioxidant defence. SOD and POD activities were identified as most significant variables responsible for shaping phenotype of Sar- and Jan-treated plants at 150 mM NaCl. Using comparative analysis of amino acid profiles, serine, threonine, proline and aspartic acid were identified as “key” amino acids in roots responsible for Sar- and Jan-mediated NaCl stress amelioration. Under both control and NaCl stress conditions, Sar exhibited higher effectiveness than Jan did. Thus, results highlight *S. muticum* and *J. rubens* as important resources which can be utilized either as “bio-stimulant” or as an starting material to identify novel components for regulating plant growth under normal and soil-saline conditions.

## Electronic supplementary material

Supplementary Tables
