# Supplementary material for: Sargassum muticum and Jania rubens regulate amino acid metabolism to improve growth and alleviate salinity in chickpea
Source: Sci Rep. 2017 Sep 5;7:10537. doi: 10.1038/s41598-017-07692-w (PMC5585251; doi:10.1038/s41598-017-07692-w)
Supplement: Supplementary file 1 — Supplementary Tables [file 41598_2017_7692_MOESM1_ESM.doc]

**Supplementary information**

### Title: *Sargassum muticum* and *Jania rubens* regulate amino acid metabolism to improve growth and alleviate salinity in chickpea

Authors: Arafat Abdel Hamed Abdel Latef, Ashish Kumar Srivastava, Hani Saber, Eman A. Alwaleed, Lam-Son Phan Tran

**Supplementary Table 1. Scores of principal components PC1 and PC2 under different treatments.** WS, Sar and Jan represent plants receiving foliar application of water, Sar and Jan extracts, respectively.

| **Treatment** | | **PC1** | **PC2** |
| --- | --- | --- | --- |
| **Control** | **WS** | -1.54 | -3.07 |
|  | **Sar** | 5.59 | 0.04 |
|  | **Jan** | 2.78 | -1.28 |
| **NaCl**  **(50 mM)** | **WS** | -3.19 | -1.39 |
|  | **Sar** | 3.83 | 0.29 |
|  | **Jan** | 2.07 | -0.22 |
| **NaCl**  **(150 mM)** | **WS** | -7.35 | 0.08 |
|  | **Sar** | -0.43 | 3.00 |
|  | **Jan** | -1.77 | 2.57 |

**Supplementary Table 2. Amino acid profiling in roots (A) and shoots (B) of chickpea seedlings under different treatments.** Using 1.5-fold as the cut-off value, amino acids showing increased levels in both Sar and Jan treatment (bold red) or only Sar (bold green), as compared with that of stressed WS plants are highlighted. WS, Sar and Jan represent plants receiving foliar application of water, Sar and Jan extracts, respectively. SD, standard deviation.

| **Roots** | **Absolute level (mg g^-1^ DW)** | | | | | | | | | | | | **Fold change** | | | |
| --- | --- | --- | --- | --- | --- | --- | --- | --- | --- | --- | --- | --- | --- | --- | --- | --- |
|  | **Control** | | | | | | **150 mM NaCl** | | | | | | **Sar control vs. WS control** | **Jan control vs. WS control** | **Sar NaCl vs. WS NaCl** | **Jan NaCl vs. WS NaCl** |
| **Amino acids** | **WS** | **SD** | **Sar** | **SD** | **Jan** | **SD** | **WS** | **SD** | **Sar** | **SD** | **Jan** | **SD** |  |  |  |  |
| **Leucine** | 1.85f | 0.078 | 8.97a | 0.121 | 3.58b | 0.005 | 2.40e | 0.040 | 2.82c | 0.114 | 2.68d | 0.010 | **4.8** | **1.9** | **1.2** | **1.1** |
| **Aspartic acid** | 1.20e | 0.087 | 5.06a | 0.225 | 2.00c | 0.007 | 0.90f | 0.100 | 2.46b | 0.006 | 1.56d | 0.005 | **4.2** | **1.7** | **2.7** | **1.7** |
| **Lysine** | 0.79f | 0.071 | 3.68a | 0.068 | 1.57b | 0.015 | 1.05e | 0.050 | 1.27c | 0.003 | 1.15d | 0.015 | **4.7** | **2.0** | **1.2** | **1.1** |
| **Histidine** | 0.94d | 0.025 | 3.68a | 0.117 | 1.14c | 0.004 | 0.90d | 0.100 | 1.59b | 0.127 | 0.98d | 0.006 | **3.9** | **1.2** | **1.8** | **1.1** |
| **Phenylalanine** | 0.58c | 0.051 | 3.22a | 0.131 | 1.14b | 0.041 | 0.75c | 0.040 | 1.00b | 0.003 | 0.98b | 0.189 | **5.6** | **2.0** | **1.3** | **1.3** |
| **Arginine** | 0.58c | 0.006 | 2.76a | 0.010 | 1.00b | 0.098 | 0.67c | 0.010 | 1.00b | 0.157 | 0.97b | 0.005 | **4.7** | **1.7** | **1.5** | **1.4** |
| **Threonine** | 0.53f | 0.020 | 2.53a | 0.020 | 1.14d | 0.005 | 0.67e | 0.061 | 1.82b | 0.003 | 1.76c | 0.020 | **4.8** | **2.2** | **2.7** | **2.6** |
| **Valine** | 0.53e | 0.023 | 2.53a | 0.025 | 1.00b | 0.055 | 0.67d | 0.021 | 0.73c | 0.008 | 0.77c | 0.049 | **4.8** | **1.9** | **1.1** | **1.1** |
| **Isoleucine** | 0.53d | 0.049 | 2.53a | 0.137 | 0.86b | 0.019 | 0.60d | 0.050 | 0.73c | 0.009 | 0.64cd | 0.012 | **4.8** | **1.6** | **1.2** | **1.1** |
| **Tyrosine** | 0.53e | 0.021 | 2.53a | 0.056 | 0.86b | 0.003 | 0.67d | 0.020 | 0.73c | 0.019 | 0.73c | 0.030 | **4.8** | **1.6** | **1.1** | **1.1** |
| **Serine** | 0.47e | 0.015 | 2.30a | 0.200 | 1.44c | 0.010 | 0.67d | 0.010 | 1.91b | 0.015 | 1.76b | 0.121 | **4.9** | **3.1** | **2.9** | **2.6** |
| **Glutamic acid** | 0.42f | 0.015 | 2.07a | 0.058 | 0.99d | 0.005 | 0.75e | 0.042 | 1.09c | 0.002 | 1.76b | 0.006 | **4.9** | **2.3** | **1.5** | **2.3** |
| **Alanine** | 0.37d | 0.025 | 1.84a | 0.104 | 0.72b | 0.013 | 0.52c | 0.010 | 0.55c | 0.004 | 0.58c | 0.004 | **5.0** | **1.9** | **1.1** | **1.1** |
| **Glycine** | 0.37d | 0.010 | 1.61a | 0.115 | 0.72b | 0.003 | 0.52c | 0.026 | 0.64b | 0.013 | 0.70b | 0.005 | **4.4** | **1.9** | **1.2** | **1.3** |
| **Proline** | 0.05e | 0.006 | 0.71a | 0.025 | 0.69a | 0.020 | 0.15d | 0.006 | 0.27b | 0.004 | 0.23c | 0.021 | **14.2** | **13.8** | **1.8** | **1.5** |
| **Methionine** | 0.15d | 0.020 | 0.46a | 0.025 | 0.29b | 0.004 | 0.22c | 0.010 | 0.27b | 0.007 | 0.30b | 0.055 | **3.1** | **1.9** | **1.2** | **1.4** |
| **Average** | **0.6** |  | **2.9** |  | **1.2** |  | **0.8** |  | **1.2** |  | **1.1** |  | **5.2** | **2.7** | **1.6** | **1.5** |

**(A)**

**(B)**

| **Shoots** | **Absolute level (mg g^-1^ DW)** | | | | | | | | | | | | **Fold change** | | | |
| --- | --- | --- | --- | --- | --- | --- | --- | --- | --- | --- | --- | --- | --- | --- | --- | --- |
|  | **Control** | | | | | | **150 mM NaCl** | | | | | | **Sar control vs. WS control** | **Jan control vs. WS control** | **Sar NaCl vs. WS NaCl** | **Jan NaCl vs. WS NaCl** |
| **Amino acids** | **WS** | **SD** | **Sar** | **SD** | **Jan** | **SD** | **WS** | **SD** | **Sar** | **SD** | **Jan** | **SD** |  |  |  |  |
| **Leucine** | 9.6d | 0.36 | 13.7a | 0.07 | 11.2c | 0.05 | 5.4f | 0.10 | 12.7b | 0.31 | 8.8e | 0.18 | **1.43** | **1.16** | **2.34** | **1.62** |
| **Aspartic** | 2.8e | 0.23 | 6.7a | 0.23 | 5.1c | 0.01 | 3.0e | 0.10 | 6.0b | 0.00 | 3.8d | 0.05 | **2.39** | **1.81** | **2.00** | **1.25** |
| **Threonine** | 2.4c | 0.10 | 4.0a | 0.30 | 3.1b | 0.01 | 1.4d | 0.04 | 3.0b | 0.00 | 2.5c | 0.10 | **1.68** | **1.31** | **2.08** | **1.74** |
| **Lysine** | 3.8c | 0.10 | 5.4a | 0.03 | 4.9b | 0.01 | 2.0d | 0.06 | 5.0b | 0.00 | 4.0c | 0.26 | **1.41** | **1.28** | **2.45** | **1.96** |
| **Phenylalanine** | 3.6d | 0.05 | 5.0a | 0.01 | 3.9c | 0.04 | 1.9f | 0.03 | 4.3b | 0.01 | 3.3e | 0.02 | **1.40** | **1.08** | **2.25** | **1.69** |
| **Valine** | 2.6d | 0.26 | 4.0a | 0.08 | 3.7b | 0.06 | 1.4e | 0.01 | 3.3c | 0.08 | 2.5d | 0.03 | **1.55** | **1.40** | **2.31** | **1.74** |
| **Serine** | 2.0d | 0.13 | 3.4a | 0.06 | 2.7b | 0.02 | 1.2e | 0.05 | 2.7b | 0.02 | 2.3c | 0.05 | **1.68** | **1.33** | **2.22** | **1.88** |
| **Arginine** | 3.6c | 0.05 | 4.7a | 0.38 | 3.9c | 0.07 | 2.0e | 0.03 | 4.3b | 0.05 | 3.0d | 0.20 | **1.30** | **1.08** | **2.12** | **1.47** |
| **Alanine** | 1.6d | 0.05 | 2.7a | 0.18 | 2.0c | 0.13 | 1.0e | 0.03 | 2.3b | 0.01 | 1.8d | 0.05 | **1.68** | **1.24** | **2.43** | **1.82** |
| **Glutamic** | 2.4c | 0.01 | 3.4a | 0.05 | 2.9b | 0.10 | 1.2d | 0.17 | 3.0b | 0.16 | 2.5c | 0.13 | **1.40** | **1.21** | **2.50** | **2.08** |
| **Histidine** | 4.0c | 0.26 | 4.8a | 0.15 | 4.4b | 0.03 | 1.9e | 0.01 | 4.3bc | 0.02 | 3.5d | 0.36 | **1.20** | **1.11** | **2.25** | **1.82** |
| **Glycine** | 2.0c | 0.10 | 2.7a | 0.04 | 2.4b | 0.15 | 1.1e | 0.02 | 2.3b | 0.02 | 1.8d | 0.13 | **1.34** | **1.21** | **2.16** | **1.62** |
| **Isoleucine** | 3.4c | 0.10 | 4.0a | 0.02 | 3.9a | 0.14 | 1.6e | 0.02 | 3.7b | 0.08 | 2.3d | 0.05 | **1.18** | **1.14** | **2.35** | **1.44** |
| **Tyrosine** | 3.4b | 0.05 | 4.0a | 0.01 | 3.9a | 0.03 | 1.5d | 0.20 | 3.3b | 0.02 | 2.5c | 0.10 | **1.18** | **1.14** | **2.22** | **1.67** |
| **Proline** | 2.0c | 0.13 | 2.5b | 0.13 | 2.2bc | 0.22 | 2.9a | 0.21 | 2.3b | 0.01 | 3.1a | 0.07 | **1.23** | **1.11** | **1.15** | **1.07** |
| **Methionine** | 1.0c | 0.05 | 1.3a | 0.05 | 1.2b | 0.03 | 0.2e | 0.01 | 1.3a | 0.01 | 0.8d | 0.02 | **1.34** | **1.23** | **5.55** | **3.13** |
| **Average** | **3.1** |  | **4.5** |  | **3.8** |  | **1.9** |  | **4.1** |  | **3.0** |  | **1.5** | **1.2** | **2.4** | **1.7** |
